# Error Correcting Mechanisms during Antisaccades: Contribution of Online Control during Primary Saccades and Offline Control via Secondary Saccades

**DOI:** 10.1371/journal.pone.0068613

**Published:** 2013-08-06

**Authors:** Harleen Bedi, Herbert C. Goltz, Agnes M. F. Wong, Manokaraananthan Chandrakumar, Ewa Niechwiej-Szwedo

**Affiliations:** 1 The Hospital for Sick Children, Toronto, Ontario, Canada; 2 Department of Ophthalmology and Vision Sciences, University of Toronto, Toronto, Ontario, Canada; 3 Department of Kinesiology, University of Waterloo, Waterloo, Ontario, Canada; University of British Columbia, Canada

## Abstract

Errors in eye movements can be corrected during the ongoing saccade through in-flight modifications (i.e., online control), or by programming a secondary eye movement (i.e., offline control). In a reflexive saccade task, the oculomotor system can use extraretinal information (i.e., efference copy) online to correct errors in the primary saccade, and offline retinal information to generate a secondary corrective saccade. The purpose of this study was to examine the error correction mechanisms in the antisaccade task. The roles of extraretinal and retinal feedback in maintaining eye movement accuracy were investigated by presenting visual feedback at the spatial goal of the antisaccade. We found that online control for antisaccade is not affected by the presence of visual feedback; that is whether visual feedback is present or not, the duration of the deceleration interval was extended and significantly correlated with reduced antisaccade endpoint error. We postulate that the extended duration of deceleration is a feature of online control during volitional saccades to improve their endpoint accuracy. We found that secondary saccades were generated more frequently in the antisaccade task compared to the reflexive saccade task. Furthermore, we found evidence for a greater contribution from extraretinal sources of feedback in programming the secondary “corrective” saccades in the antisaccade task. Nonetheless, secondary saccades were more corrective for the remaining antisaccade amplitude error in the presence of visual feedback of the target. Taken together, our results reveal a distinctive online error control strategy through an extension of the deceleration interval in the antisaccade task. Target feedback does not improve online control, rather it improves the accuracy of secondary saccades in the antisaccade task.

## Introduction

Saccades are stereotypical eye movements characterized by the main sequence, which describes the linear relationship between saccade amplitude and peak velocity/duration [1]. Despite the stereotypical saccade pattern, there is a degree of variability in motor performance that is inherent in eye movements. Saccades are susceptible to errors which can arise during sensory coding, sensorimotor transformation and/or motor execution [2]. In this paper, we investigated the error correction processes in antisaccades, which are volitional eye movements that require suppression of a reflexive saccade towards a visual target, and generation of a saccade towards a location that is the mirror opposite of where the target was presented [3].

The antisaccade task has been utilized extensively in previous experiments as a model for the investigation of volitional saccades [4], [5], [6], [7]. The successful execution of the antisaccade requires two higher cognitive processes: inhibition of a reflexive saccade and inversion of the saccade vector in order to make an eye movement to the mirror location. Most studies in the literature have focused on the first process – the ability to inhibit a reflexive saccade. The focus of our study is on the second process – saccade vector inversion which contributes to antisaccade inaccuracy [8]. In other words, vector inversion is an internally generated error that introduces uncertainty about target location. Thus, we investigated whether, and to what extent, this internally generated representation and its associated error can be amended during the ongoing eye movement. In general, saccade accuracy can be maintained via two control mechanisms: online (i.e., in-flight corrections during the ongoing primary saccade) and offline (i.e., programming of a secondary eye movement). Details of these two error correction mechanisms are discussed below.

### I. Online saccade control

Given the relatively short duration of saccades, older models of oculomotor control proposed that in-flight modifications of saccadic trajectory (i.e., online control) could not occur [9]. Subsequently, Robinson [10] proposed a particularly influential model which included internal feedback loops that enabled in-flight modulation of saccade trajectory. According to this model, efference copy provides an internal feedback signal, which can be subtracted from the desired eye position to compute a dynamic motor error signal that can be used to correct the ongoing eye movement. While most studies considered the efference copy of the motor command as the sole source of the error signal for online control [11], [12], [13], other studies have suggested that visual feedback can also affect the dynamics of the ongoing saccades [14], [15]. However, the view that visual feedback can be used to change the kinematics of the ongoing saccade has been challenged by short saccade duration (∼50–80 ms for 15–20° saccades) [1], long latencies in the visual system (∼80–100 ms) [13], [16], [17], and reduced visual sensitivity during saccades. For example, visual suppression starts ∼50 ms prior to saccade initiation, reaches maximum around the onset of the saccade and is reduced as the saccade progresses [18], [19], [20]. Thus, the issue whether visual feedback can be used online to modify saccade trajectory remains controversial.

The potential role of online control in the antisaccade task was examined in only two studies [17], [21] with conflicting conclusions. Heath and colleagues [21] examined online control using a regression analysis. According to this technique, the spatial position of the eye at different time points within the saccade trajectory is used to determine the proportion of explained variance in endpoint eye position (R^2^). They reasoned that if participants engage in feedback-based online control, then initial errors in saccade amplitude will be detected and ameliorated during the ongoing movement. Therefore, online control can be inferred from lower R^2^ values during the middle and later stages of the saccade trajectory. Alternatively, higher R^2^ values combined with larger endpoint error indicate a diminished ability to make correction online because errors in movement programming that are evident early during the trajectory remain uncorrected at the end of the movement. Using the regression analysis, Heath et al. [21] found significantly higher R^2^ values in the antisaccade task in comparison to the reflexive saccade task, and concluded that online control plays a lesser role in antisaccades than in reflexive saccades. These conclusions are in contrast to those reported by Xu-Wilson et al. [17]. These authors showed that antisaccades are also amenable to online trajectory control in a manner analogous to reflexive saccades. Specifically, it was found that despite the introduction of a transcranial magnetic stimulation (TMS)-induced perturbation during the antisaccade, the eyes were guided close to the target location. Thus, the induced perturbation error was compensated with subsequent motor commands, indicating that oculomotor control of antisaccades emulates the internal feedback model that was originally proposed by Robinson [10] for saccades. Given the contradictory results regarding online control of antisaccades, the first objective of this study was to examine online control of antisaccades by investigating eye movement dynamics. Specifically, we examined whether the lengthening of the saccade deceleration interval in the antisaccade task, which has been previously reported in literature [3], [22], contributes to improved end-point accuracy. We examined the relation between the extended duration of deceleration phase and movement accuracy, because while this extended deceleration has been documented previously, its functional significance has not been determined. Another reason for examining the deceleration phase is that visual sensitivity is reduced at saccade initiation; however, the suppression is reduced as the trajectory evolves [19]. If visual feedback can be used to modify the trajectory of an ongoing saccade, the correction would have to be implemented late in the trajectory, i.e., during the deceleration phase. Thus, we hypothesized that the extended deceleration duration would be related to improved accuracy, which most likely reflects an online error correction process.

### II. Offline control: secondary saccades

Given the short duration of saccades, the online error correction mechanism is limited in terms of the size of the correction that can be implemented. If the error is too large or the direction of the saccade is incorrect, the oculomotor system must rely on a second eye movement to correct the remaining error. The kinematics of secondary saccades generated in a reflexive saccade task have been studied extensively. The amplitude of secondary saccades typically represents 10% of primary saccade amplitude [23] and the latency of secondary saccades ranges between 100–250 ms [24]. In contrast, secondary saccades generated following antisaccades have not been studied in detail. Hallet [3] reported shorter latency and larger amplitude of secondary saccades in the antisaccade task in comparison to the reflexive saccade task. He suggested that secondary saccades in the antisacccade task were correcting for the error remaining after the primary saccade and were likely programmed based on extraretinal information derived from the efference copy. These findings were extended by Krappmann and colleagues [8] who showed that the secondary saccades in the antisaccade task compensated significantly, but not completely, for the primary saccade endpoint amplitude error. The second objective of our study was to characterize the kinematics and the contribution of secondary saccades to antisaccade end-point accuracy. Considering the large inaccuracy and higher variability of antisaccades, we hypothesized that secondary saccades would have a major role in reducing the final error in the antisaccade task, which would be enhanced by the presence of visual feedback.

## Materials and Methods

### I. Participants

Fourteen visually normal participants (age: 27±6 years; 8 females) were recruited. All participants had normal or corrected-to-normal visual acuity of 20/20 or better in each eye. The study was approved by Research Ethics Board of The Hospital for Sick Children and all protocols adhered to the tenets of the Declaration of Helsinki. Written informed consent was obtained from each participant prior to participation.

### II. Apparatus

The visual stimulus was a red laser dot (visual angle ≈0.2°) projected onto a translucent back-projection screen which was located 80 cm in front of the participant. The stimulus was generated using a laser-beam galvanometer (GSI Group©, USA), with a bandwidth of 5000 Hz. Eye movements were recorded binocularly at 200 Hz using an infrared video-based pupil/iris tracking system (C-ETD Eye Tracker, Chronos Vision, Berlin, Germany). This system has a maximum resolution of 6 min of arc over a range of ±20° and a linearity of <0.5% for both horizontal and vertical eye movements. Prior to data collection, a horizontal and vertical calibration was performed for each eye using fixation targets at five locations: 0° and ±10° horizontally and vertically. The real-time eye position data from the eye tracker were differentiated using a five-point quadratic polynomial Savitzky-Golay smoothing filter to yield the online eye velocity profile. The online eye velocity signal was then used to control target presentation using a threshold velocity of 50°/s. Specifically, when the velocity threshold was reached, the target was switched off in the reflexive saccade task or illuminated at the spatial goal in the antisaccade task (on 50% of the trials). Eye position data were stored on a computer for offline analysis.

### III. Experimental design and procedure

Participants were seated in a dimly lit room with their head stabilized with a chin rest while fixating on a red laser dot presented straight ahead. After a variable delay of 1.5–3 s, the target was displayed at eye level, at one of six locations (±19°, ±22° or ±25° along the horizontal axis) in random order. Targets were presented at 19°, 22° and 25° to elicit saccades with duration >60 ms in order to maximize the opportunity for visual feedback to be used in the local feedback loop. Participants were instructed to move their eyes to fixate the target (reflexive saccade task) or to make an eye movement of equal amplitude and opposite direction to target location (antisaccade task) as accurately as possible (speed of responding was not emphasized in the instructions to the participants). The reflexive saccade task and the antisaccade task were conducted in separate blocks, with presentation order randomized across study participants.

**Figure 1:**
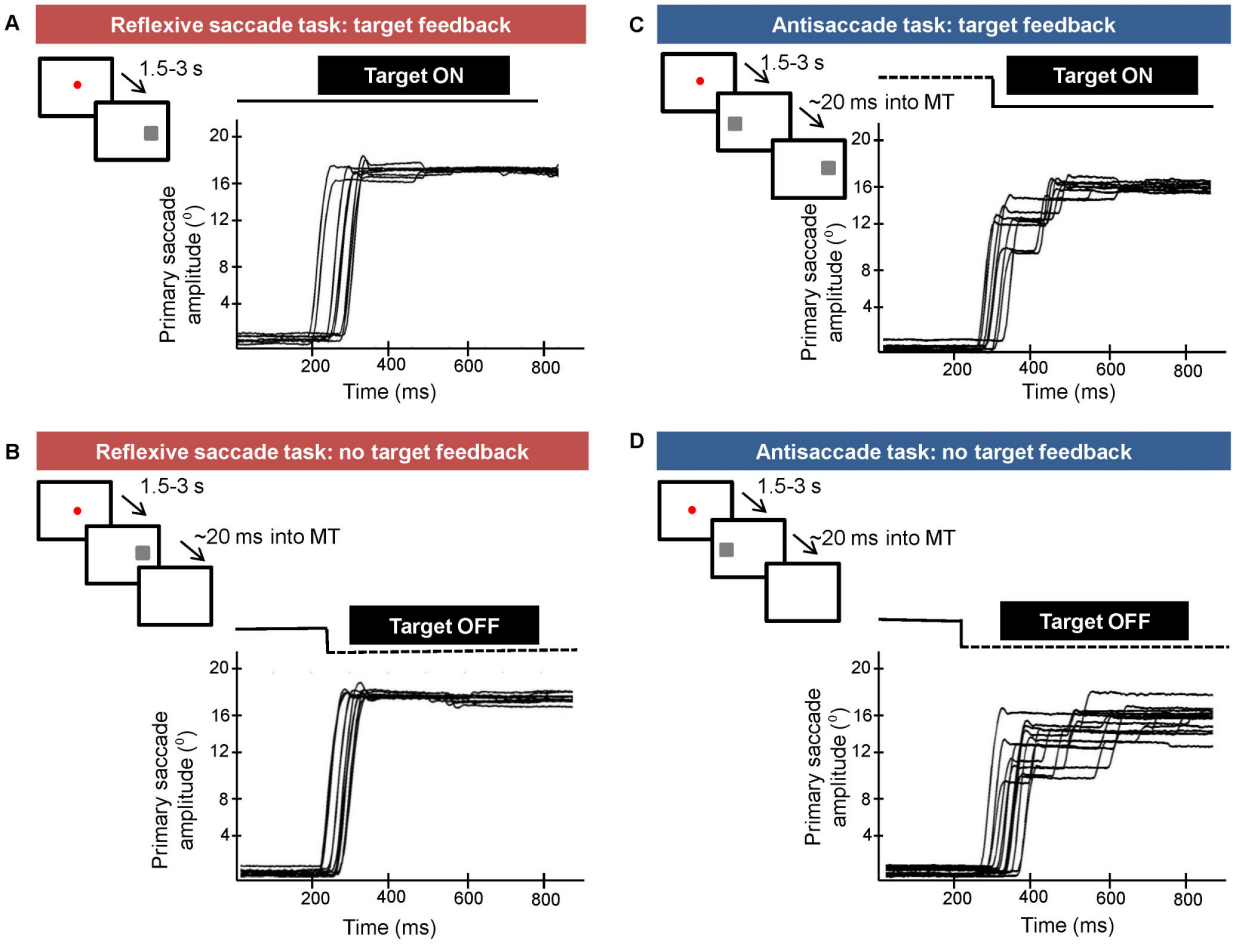
Schematic illustration of the experimental procedure and representative eye movement data from one subject for targets presented at +19° in the reflexive saccade task and −19° in the antisaccade task. (A) In the Reflexive Saccade - target feedback condition, the target was illuminated for the duration of the trial. (B) In the Reflexive Saccade – no target feedback condition, the target was extinguished as soon as real-time eye velocity signal was >50°/s (≈ within the first 20 ms into the saccadic movement time, MT). (C) In the Antisaccade – target feedback condition, the target was illuminated at the expected primary saccade location (i.e., mirror-opposite to the initial target location as soon as eye velocity signal was >50°/s. (D) In the Antisaccade task – no target feedback condition, the target was extinguished as soon as eye velocity signal was >50°/s.

Figure 1A shows a schematic illustration of the experimental paradigm and the representative eye movement data from one participant. Visual feedback of the target was provided in randomized fashion on 50% of the trials in the reflexive saccade task and antisaccade task. Participants performed a total of 120 trials in the reflexive saccade task. During feedback trials in the reflexive saccade task, the target remained visible throughout the duration of the trial (i.e., target feedback was present both during the ongoing saccadic eye movement as well as at the end of the primary saccade, Figure 1A). In the remaining trials, the target was extinguished as soon as the eye velocity signal reached >50°/s (≈ within the first 20 ms of the saccadic eye movement, Figure 1B).

In the antisaccade task, each trial began with a central fixation dot and the target was illuminated after a variable delay of 1.5–3 s, at which point the fixation dot was extinguished (no gap paradigm). Participants were instructed to look in the mirror-opposite direction and amplitude to where they saw the target. In target feedback trials, as soon as the real-time eye velocity signal was >50°/s, the target was illuminated at a location mirror-opposite to where the initial target was presented (i.e., the antisaccade target was shown at the location where the antisaccade was supposed to land). The target remained visible for the entire duration of that trial (Figure 1C). In the no feedback trials, the initial target was extinguished as soon as the eye velocity was >50°/s. Participants performed a total of 180 trials in the antisaccade task. Our design included 30% more trials in the antisaccade task in anticipation of a higher directional error rate in this condition (i.e., trials in which the antisaccade was initiated in the wrong direction).

### IV. Data analysis

Eye movement data were analyzed using a custom-written C++ program. Eye velocity data were obtained by differentiating position data using a five-point quadratic polynomial Savitzky-Golay smoothing filter. Saccades were marked using a velocity threshold of 20°/s for primary saccades and a velocity threshold of 15°/s for secondary saccades. All trials were inspected visually to ensure that primary and secondary saccades were identified correctly by the program. Any trials where the primary saccade was initiated in the wrong direction (i.e., saccade towards the target in the antisaccade task) were excluded from the analysis (10% of total antisaccade trials).

Primary saccades were rejected if their latency was >500 ms or peak velocity was <100°/s. These exclusion criteria are commonly used in saccade studies to exclude trials with delayed saccade initiations due to lapses in attention or saccades with atypical velocity profiles, for example, glissades [25], [26], [27]. These exclusion criteria led to rejection of 2.5% of primary saccades in the reflexive task and 9% in the antisaccade task.

Secondary saccade latency was defined as the interval from the end of the primary saccade to the initiation of the next saccadic movement. Secondary saccades were rejected if their latency was >300 ms. Previous studies have reported that secondary, corrective saccades are usually initiated with an average latency of 200 ms, but the distribution usually includes saccades with latencies of up to 300 ms [24], [28], [29].

#### i. Descriptive Statistics

Mean saccade latency, duration, amplitude, gain (saccade amplitude/target amplitude) and peak velocity were calculated for each experimental condition. Preliminary analysis showed that there was no difference for any saccade metrics between leftward and rightward movements for each target location. Therefore, trials of the same target eccentricities were combined in the subsequent analysis. All outcome measures for the primary saccades were submitted to repeated-measures ANOVA with 3 factors: Task (saccade, antisaccade), Target Feedback (feedback, no feedback) and Target Amplitude (19°, 22°, 25°).

#### ii. Online Control - Primary Saccades

Online control of saccades was examined by calculating the duration of acceleration and deceleration intervals, and the skewness ratio (i.e., duration of deceleration interval/duration of acceleration interval). Since we expected that the duration of these intervals might be affected by target location, these outcome measures were submitted to repeated-measures ANOVA with 3 factors: Task (reflexive saccade, antisaccade), Target Feedback (feedback, no feedback), and Target Amplitude (19°, 22°, 25°).

Correlation analysis was conducted to examine whether the duration of deceleration interval was associated with saccade endpoint error. Pearson's correlation coefficients relating the error remaining at the end of the primary saccade with the duration of the deceleration period were calculated for each participant, task and target feedback condition. In order for the regression analysis to provide a more robust result, the regression was conducted on data combined across target locations. A strong negative correlation would indicate that the amplitude error of the primary saccade was compensated through a lengthening of the deceleration interval (i.e., slope of −1 would indicate perfect error compensation).

The correlation coefficients were transformed into Fisher z-scores, which were submitted to repeated-measures ANOVA with 2 factors: Task (reflexive saccade, antisaccade) and Target Feedback (feedback, no feedback).

#### iii. Offline Control - Secondary Saccades

To examine the contribution of secondary saccades to the final end-point accuracy in the reflexive and antisaccade tasks, we first computed the frequency of secondary saccades in each task. Pearson's chi-square statistic was used to determine if the difference between tasks was significant.

A correlation analysis was conducted to examine the extent to which secondary saccades corrected for the error remaining at the end of the primary saccades. The end-point error was calculated as the percent difference between the target location and primary saccade amplitude (i.e., % under- or overshoot). The amplitudes of the secondary saccades were normalized, i.e., expressed as % of the target eccentricity to compare error corrections occurring at different target amplitudes. Pearson's correlation coefficients were calculated relating primary saccade error and secondary saccade amplitude. Our preliminary analysis showed that for each target location, there was no difference in any primary saccade and secondary saccade metrics between leftward and rightward movements. Therefore, our correlation analysis was performed using the absolute constant error of saccade endpoint in the direction of the primary movement (i.e., horizontal direction). A high correlation indicates that the secondary saccade compensated for primary saccade end-point error (i.e., slope of 1 would indicate perfect error compensation). The correlation coefficient was calculated for each participant, task and target feedback condition and transformed into Fisher z-scores which were submitted to repeated-measures ANOVA with 2 factors: Task (saccade, antisaccade) and Target Feedback (feedback, no feedback).

Additional analysis was carried out to further investigate the contribution of extraretinal and retinal feedback in programming of secondary eye movements in the reflexive saccade and antisaccade tasks. In the first analysis the effect of Target Feedback on the frequency of secondary saccades was examined separately in each task using Pearson's chi-square statistic. When target feedback was not available, secondary saccades must have been programmed based on extraretinal information.

In the second analysis, secondary saccades were divided into two groups based on their latency. Early-onset secondary saccades with latency <80 ms are most likely initiated based on extraretinal information, whereas late-onset with latency ≥80 ms can be initiated based on retinal feedback. A cut-off value of 80 ms was used to ensure that there was enough time for retinal feedback to influence the programming of a secondary saccade. This time is based on the afferent and efferent delays in processing of visual information to generate an oculomotor command [30], [31], [32]. In addition, the lowest saccade latencies to visual targets are found in this range (express saccades) [33], [34]. Consequently, secondary saccades with latencies <80 ms are more likely to be pre-programmed based on extraretinal sources of feedback. The frequency of early-onset and late-onset secondary saccades in the reflexive saccade and antisaccade tasks was compared using Pearson's chi-square statistic. Subsequently, we also examined the amplitude and peak velocity of the early and late-onset secondary saccades in each task when visual feedback was on and off.

All statistical analysis was performed using the SAS 9.2 software package (Cary, NC). Significant main effects from all ANOVAs were analyzed further using Tukey-Kramer post-hoc tests to reduce the possibility of Type I error due to multiple comparisons.

## Results

### I. Primary Saccades

Table 1 shows the summary data for primary saccade kinematic outcome measures. Primary saccades were predominately hypometric (undershoot error, i.e., Gain<1). In the reflexive saccade task, 71% of saccades landed short of the target (undershoot error = 1.80±1.53° [8% undershoot]; overshoot error = 1.14±1.12° [4% overshoot]). In the antisaccade task 82% of saccades undershot the target (undershoot error = 6.61±4.33° [28% undershoot]; overshoot error = 2.19±1.72° [11% overshoot]). Pearson chi square analysis confirmed that the difference in the number of hypometric saccades between tasks was significant (χ^2^_(df = 1)_ = 68.75, p<0.0001). These large errors in the antisaccade task were due to the lack of amplitude scaling with target distance. In particular, as shown in Table 1, the mean saccade amplitude in the antisaccade task was 17° when the targets were presented at 19°, 22° or 25°. This is in striking contrast to the reflexive saccade task where amplitude was clearly scaled to each target location (19° target: 17.85±0.91°; 22° target: 21.22±1.19°; 25° target: 24.7±1.29°).

**Table 1:** Means and standard deviations of primary saccade kinematics in the reflexive saccade task and the antisaccade task for the different target locations (19°, 22°, 25°) when the target was present (ON) or not (OFF).

	Reflexive Saccade Task						Antisaccade Task						
Target step	19°		22°		25°		19°		22°		25°		
Target condition	OFF	ON	OFF	ON	OFF	ON	OFF	ON	OFF	ON	OFF	ON	Statistical results
Latency (ms)	186±21	191±24	193±19	189±22	198±19	196±25	287±34	289±31	287±25	288±22	299±23	300±27	*F(1,13) = 162.07; p<0.0001
Duration (ms)	67±8	67±8	76±11	77±11	88±12	87±13	77±14	78±14	79±16	78±15	81±16	79±16	∧F(2,26) = 88.08; p<0.0001
Amplitude (°)	17.82±0.91	17.88±0.95	21.19±1.20	21.26±1.22	24.67±1.22	24.47±1.40	16.96±2.43	17.08±2.30	17.39±3.02	17.27±2.80	17.65±2.47	17.26±2.89	∧F(2,26) = 222.44; p<0.0001
Peak velocity (°/s)	396.0±49.01	398.48±52.93	416.77±60.31	412.54±56.99	424.45±58.82	423.79±61.15	320.76±45.50	319.91±40.90	320.10±40.06	322.45±42.05	321.77±35.47	319.33±45.94	∧F(2,26) = 14.18; p<0.0001
Primary saccade amplitude variability (°)	1.06±0.40	1.20±0.37	1.51±0.72	1.42±0.53	1.37±0.44	1.44±0.44	3.82±1.00	3.62±1.35	3.66±1.35	3.52±1.15	3.64±1.15	3.57±0.99	∧F(2,26) = 3.78; p = 0.03

* Antisaccade latency was significantly longer compared to reflexive saccades.

∧ Reflexive saccade duration, amplitude, peak velocity and precision were scaled with target step. In contrast, antisaccade kinematics were similar across all target steps.

The effect of Target Feedback was not significant for any of the outcome measures in either the reflexive saccade or the antisaccade task, suggesting that visual feedback did not affect the primary saccade kinematic measures in either task.

### Online Control during Primary Saccades

#### Acceleration/Deceleration Interval (Skewness) Ratio

Saccades in both tasks (reflexive saccade and antisaccade) exhibited a rightward skew of the velocity profile, which was due to the extension of the deceleration interval. The mean skewness ratio (i.e., duration of deceleration interval/duration of acceleration interval) was significantly higher for antisaccades (2.12±0.52) than for reflexive saccades (1.76±0.41) (F_(1,13)_ = 19.6, p = 0.0007). For reflexive saccades, the duration of acceleration interval was similar across the 3 target amplitudes (28±3 ms); however, the deceleration interval increased with target amplitude (19° target: 40±6 ms; 22° target: 48±10 ms; 25° target: 58±11 ms). For antisaccades, the acceleration and deceleration intervals were similar for the 3 target amplitudes (acceleration interval: 25±3 ms, deceleration interval: 53±13 ms). No significant effect of Target Feedback was found for the duration of deceleration phase (F_(1,13)_ = 0.75, p = 0.403).

#### Relationship Between Duration of Deceleration Interval and Accuracy

**Figure 2:**
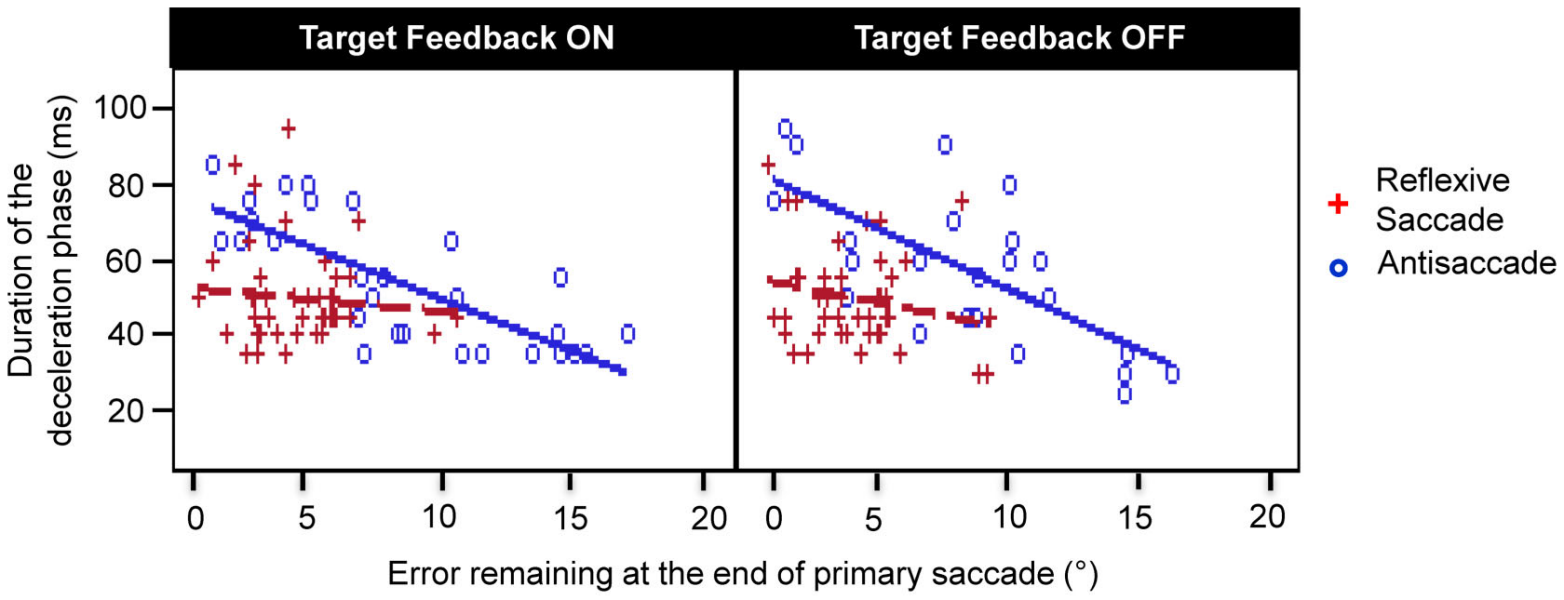
Representative data from one participant showing correlation between the duration of the deceleration phase and the end-point error of primary saccades for the reflexive saccade task and the antisaccade task. A stronger negative correlation was observed in the antisaccade task as compared to the reflexive saccade task (p<0.0001).

The relationship between the duration of deceleration interval and primary saccade endpoint error was investigated using Pearson's correlation coefficient. Figure 2 shows representative data from one participant. In the antisaccade task, there was a stronger negative correlation between the deceleration interval and endpoint error (i.e., a longer deceleration interval was associated with smaller end-point error) as compared to the reflexive saccade task. Specifically, across all participants, the mean Pearson correlation coefficient was −0.75±0.22 (corresponding z-score −1.09±0.41) in the antisaccade task, as compared to −0.36±0.41 (corresponding z-score 0.31±0.34) in the reflexive task (F(_1,13)_ = 205.87, p<0.0001). No significant effect of Target Feedback was found (F_(1,13)_ = 3.02, p = 0.106).

### II. Secondary Saccades

**Figure 3:**
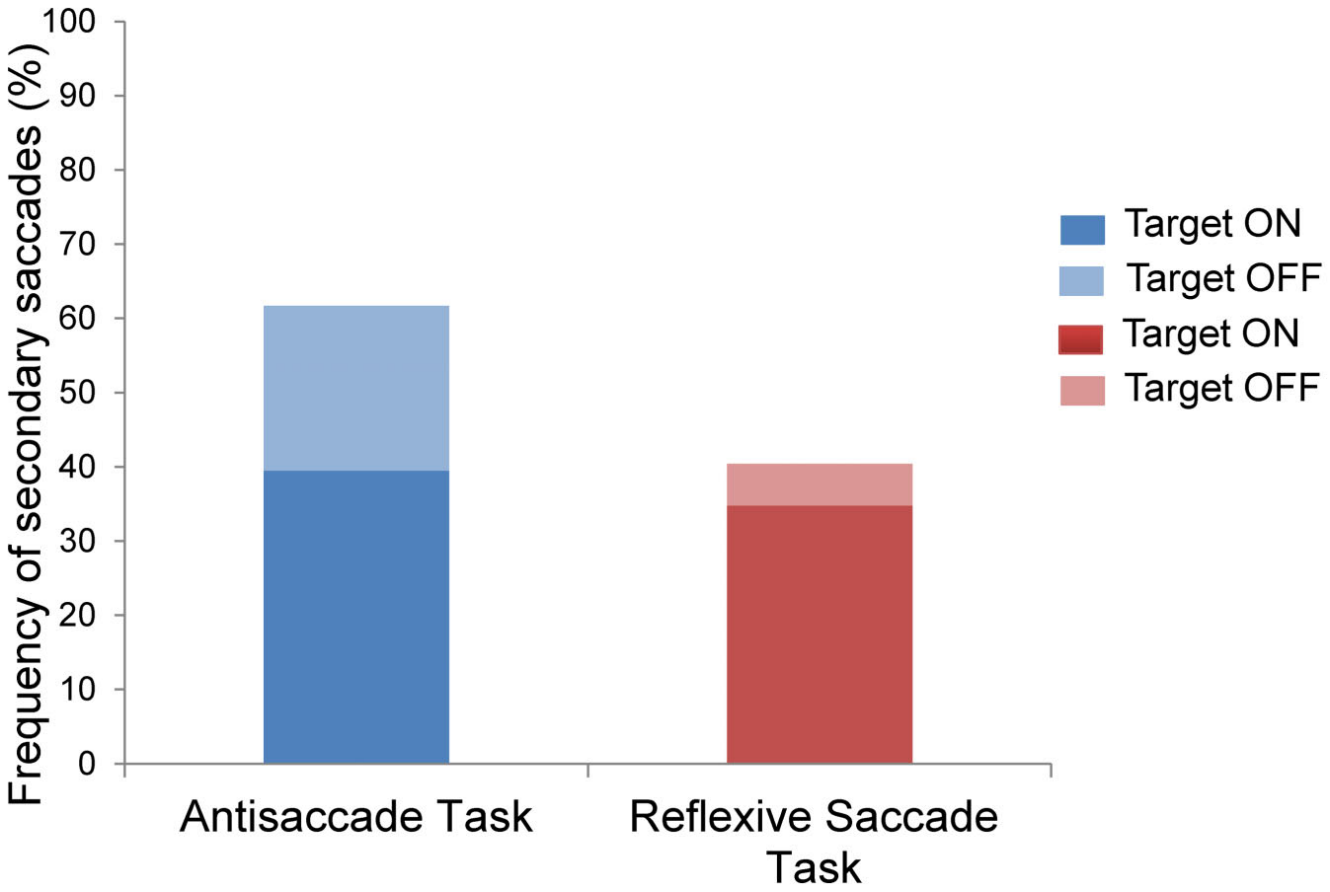
Frequency of secondary saccades in the antisaccade and reflexive saccade tasks. Secondary saccades were generated more frequently in the antisaccade task, which was specifically evident when target feedback was not available (p<0.0001).

Overall, participants generated significantly fewer secondary saccades in the reflexive saccade task (41%) as compared to the antisaccade task (61%) (χ^2^_(df = 1)_ = 160.06, p<0.0001). As illustrated in Figure 3, secondary saccades were initiated more frequently when target feedback was available in both the reflexive saccade task (feedback: 34.8%, no feedback: 5.6%; χ^2^_(df = 1)_ = 526.44, p<0.0001) and the antisaccade task (feedback: 39.5%, no feedback: 22.2%; χ^2^_(df = 1)_ = 194.15, p<0.0001). The between-tasks difference in frequency of secondary saccades was mainly due to a greater number of these saccades in the antisaccade task no feedback condition.

### Offline Control via Secondary Saccades

**Figure 4:**
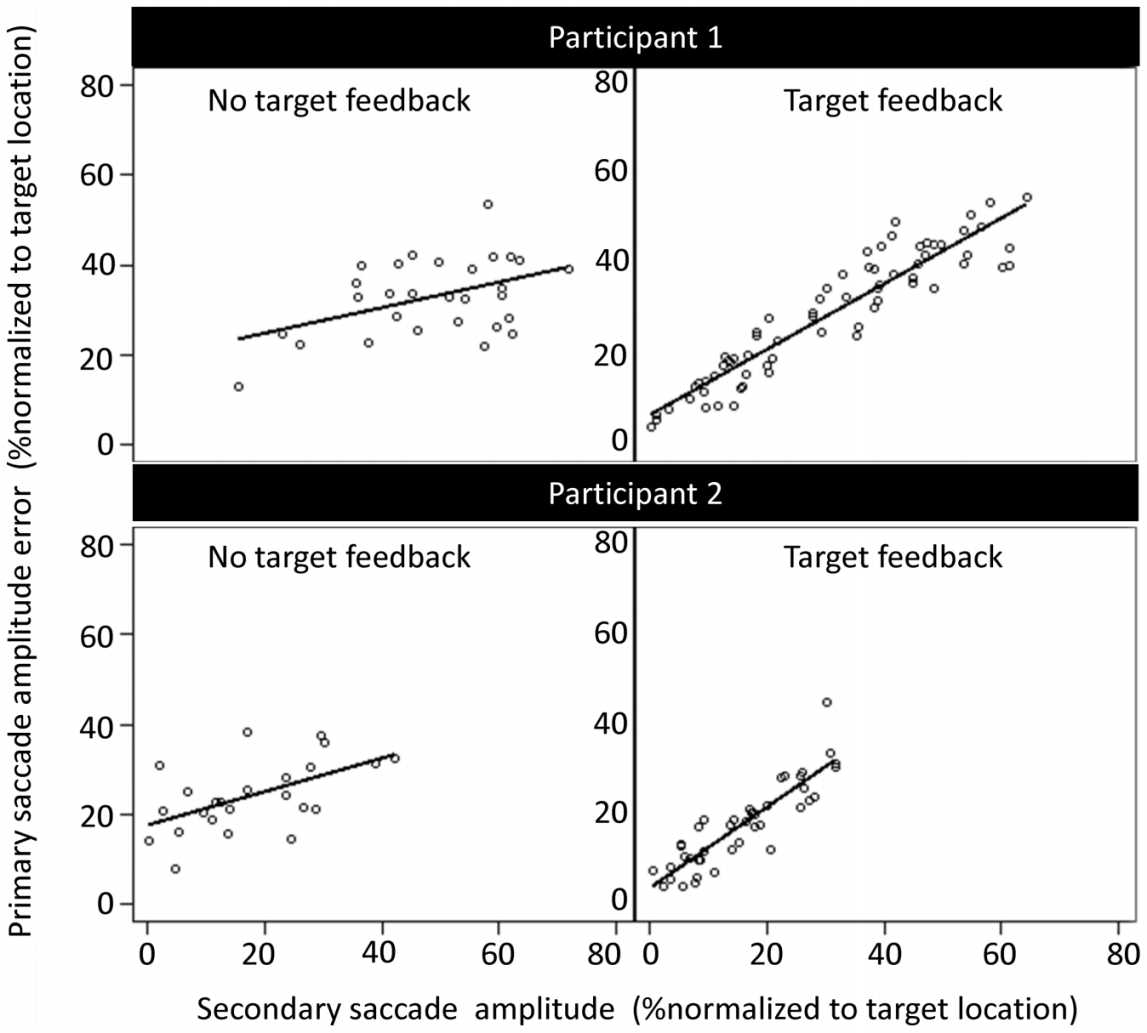
Representative data from two participants showing correlation between primary saccade end-point error and secondary saccade amplitude (normalized to target location) for the antisaccade task with no target feedback condition (left panel) and with target feedback condition (right panel). Secondary saccades corrected for the amplitude error remaining at the end of the primary saccade to a greater degree when target feedback was available (p<0.0001).

We examined the extent to which secondary saccades corrected for the remaining amplitude error in the antisaccade task by calculating the Pearson's correlation coefficient between primary saccade endpoint error and secondary saccade amplitude. Figure 4 illustrates the relation between the error remaining at the end of the primary saccade (i.e., the % under or overshoot) and secondary saccade amplitude (both normalized to target location) for two typical participants. Statistical analysis confirmed a significant effect of Target Feedback (F(_1,13)_ = 27.7, p = 0.0002). With target feedback, the mean Pearson's correlation coefficient averaged across participants was 0.77±0.23 (z-scores 1.21±0.50), while without target feedback, the correlation coefficient was 0.40±0.49 (z-score 0.61±0.66).

### III. Role of Extraretinal and Retinal Feedback

#### Frequency

The frequency of early-onset secondary saccades (i.e., onset was <80 ms after the termination of the primary saccade) was approximately six-fold higher in the antisaccade task (17.5%) as compared to the reflexive saccade task (2.7%). Target Feedback did not affect the frequency of early onset secondary saccades in either task. In contrast, there was a significant effect of Target Feedback on the frequency of late-onset secondary saccades in both the reflexive task (χ^2^_(df = 1)_ = 10.86, p = 0.001) and the antisaccade task (χ^2^(_df = 1)_ = 16.72, p<0.0001). Participants generated a seven-fold higher frequency of late-onset secondary saccades in the reflexive task in the presence of target feedback (feedback 86.85%, no feedback 13.15%), while there was only a two-fold increase in the antisaccade task (feedback 66.7%; no feedback 33.4%).

#### Latency of Early vs. Late Onset Secondary Saccades

The latency of early-onset secondary saccades was not influenced by the presence of target feedback in the reflexive task (feedback 46.50±20.55 ms, no feedback 60.00±15.81 ms) or in the antisaccade task (feedback 46.80±15.97 ms, no feedback 46.94±16.81 ms). On the other hand, the late-onset secondary saccades were initiated earlier with target feedback present in both the reflexive task (feedback 178.95±43.18 ms, no feedback 195.25±69.67 ms) and the antisaccade task (feedback 165.77±52.70 ms, no feedback 185.58±62.17 ms, [F_(1,13)_ = 106.45, p<0.0001]).

#### Amplitude of Early vs. Late Onset Secondary Saccades

The amplitude of early-onset secondary saccades in the antisaccade task was significantly larger (6.20±2.70°) compared to the late-onset secondary saccades (4.74±2.57°; F_(1,12)_ = 20.83, p = 0.0006). Similarly, secondary saccade amplitude in the reflexive task also seemed to follow the same trend that was observed in the antisaccade task, with larger mean amplitudes in the early-onset saccades as compared to the late-onset saccades regardless of target feedback. However, given the lower frequency of early-onset secondary saccades in the reflexive task, a similar statistical analysis for mean latency and mean amplitude of secondary could not conducted.

The presence of visual feedback influenced the extent of correction in the antisaccade task (F(_2,26)_ = 2.18, p = 0.027). Specifically in case of late-onset secondary saccades, amplitude and peak velocity increased for larger target eccentricities (19° target: 3.6±1.2° PV: 139±46°/s; 22° target: 4.7±1.4°, PV: 171±44°/s; 25° target: 5.8±1.7°, PV: 195±44°/s). When target feedback was not available, secondary saccade amplitude and peak velocity were not influenced by target location in the antisaccade task (19° target: 4.6±1.6° PV: 158±50°/s; 22° target: 4.5±1.8°, PV: 158±56°/s; 25° target: 5.1±1.6°, PV: 170±55°/s).

## Discussion

The main goal of our study was to examine the contribution of online and offline control mechanisms to end-point accuracy in the antisaccade task. This task can provide insight into the oculomotor mechanisms that are involved in correcting internally generated errors during the vector inversion process. In other words, in the antisaccade task the original target location has to be inverted to program a correct saccade vector to a mirror spatial location. These oculomotor errors can be corrected online (i.e., during the ongoing saccade) and/or offline by initiating a secondary, corrective saccade. In the case of antisaccades, we found that: (1) the extension of antisaccade deceleration interval is a feature of online control that was associated with improved accuracy; (2) target feedback presented at the spatial goal of the antisaccade does not alter the dynamics of the ongoing eye movement; (3) generation of secondary saccades is an important control strategy that improves endpoint accuracy of antisaccades, which is further enhanced by, visual feedback.

### I. Online control of antisaccades

In agreement with previous studies, we found that antisaccades had longer latency, lower accuracy and greater variability compared to reflexive saccades [3], [8], [22], [35], [36], [37]. Furthermore, consistent with the previous literature [3], [22], we observed that the velocity profile of antisaccades was highly asymmetric due to extension of the deceleration phase. Importantly, this extension of the deceleration phase was associated with reduced antisaccade end-point error. Our findings are analogous to what has been reported previously for upper limb reaching movements [38], [39], [40], [41]. Specifically, it has been proposed that online modifications of the limb trajectory are made in the deceleration phase of the reaching movement. However, unlike reaching movements in which the deceleration phase is extended when target feedback is available, our results show that visual feedback during eye movements did not affect the duration of deceleration phase. We propose that the increased time spent in the deceleration period during antisaccades is indicative of an oculomotor online error control strategy, which allows small in-flight modifications of saccadic trajectory. Furthermore, we found that visual feedback of the target did not affect any other saccade kinematic measures, thus, the main signal to guide these in-flight modifications most likely comes from the efference copy, as was originally proposed by Robinson [10].

The data from our study are in agreement with Xu-Wilson and colleagues [17]. These authors used TMS to perturb saccadic trajectory and found that, despite an induced in-flight error, the eyes landed close to the target location in the antisaccade task. These findings suggest that once an antisaccade is initiated, the ongoing eye movement is controlled by internal feedback that is characteristic of an internal forward model as proposed Robinson [10]. According to this model, the saccadic pulse generator receives a dynamic update of the current eye position, which then allows online modification of the saccadic trajectory to improve accuracy and reduce endpoint variability.

In contrast to our results, Heath et al. [21], [42] reported poorer online control of antisaccades compared to reflexive saccades. Their conclusions were based on a regression analysis technique, which was originally developed to examine the control of upper limb reaching movements [43], [44]. It is possible that the regression analysis may not be a sensitive technique for detecting in-flight modulations of eye movements, because saccade duration is relatively short in comparison to reaching movements. Thus, online control in the oculomotor system is inherently limited and will only afford small modifications of saccade trajectory, which might not be evident in the regression analysis. Additionally, from a neurophysiological standpoint, it seems intuitive that the internal control of antisaccades and reflexive saccades should be similar once the saccade is initiated. In particular, once the motor command reaches the output centers, the feedback controller could utilize a similar forward model to monitor and maintain the accuracy of the ongoing eye movements.

Theoretically, input to the forward feedback model could come from several sources, including the efference copy of the oculomotor command [10], [45], [46], visual reafference [15], [47] and proprioception from extraocular muscles [48], [49], [50]. We examined the role of vision by investigating whether target feedback enhances online control. Specifically, we compared primary saccade dynamics on trials in which no target feedback was available with those in which feedback was provided during the execution of the saccade (i.e., the target was illuminated at the actual goal of the task during the antisaccade). The results from Gaveau et al. [47] and West et al. [15] showed that visual feedback can be used for online control for large saccades with durations >50 ms. Thus, we presented targets at 19°, 22° and 25° to elicit saccades with duration >60 ms in order to provide the best opportunity for visual feedback to be used in the local feedback loop. Our results show that target feedback did not enhance online control of saccadic trajectory in either the antisaccade or reflexive saccade tasks. Specifically, we observed no effect of visual feedback on saccade accuracy, precision, peak velocity, the duration of deceleration, or the extent to which the extension of the deceleration phase was correlated with saccade endpoint error. Therefore, results from our study corroborate the previously held belief that visual feedback likely plays a minor role in the online control of saccades [11], [13], [17].

### II. Secondary saccades as a compensatory “offline” error correction strategy

Considering that antisaccades are inaccurate [4], [5], [8], [35], [36], [37], [51] and that the online control of saccade trajectory is limited, we also examined the role of secondary saccades as an “offline” error correction strategy. Our results showed that secondary saccades were generated more frequently in the antisaccade task compared to the reflexive saccade task. Our findings are in contrast to the observation made by Hallett [3] who reported “delayed, very inaccurate, primary [anti]saccades, which were more usually not followed by secondary saccades.” On the other hand, Krappmann et al. [8] reported a high frequency of secondary saccades in the antisaccade task (∼66%), which is consistent with our results. Both studies by Hallett and Krappmann et al. used a similar antisaccade task and it is not immediately apparent why there is a discrepancy in their findings for the frequency of secondary saccades.

We postulate that the increased frequency of secondary saccades in the antisaccade task is an important strategy to compensate for their inherent inaccuracy. The relative contributions of retinal feedback and extra-retinal signals to the generation of corrective eye movements have been studied extensively in the case of reflexive saccades [24], [28], [52]. Briefly, extraretinal feedback provides an important contribution to the generation of secondary saccades for targets at large eccentricities (>15°) or if the primary saccade is very hypometric (error >3°) [9]. Retinal feedback provides a very important contribution to the generation of secondary saccades following reflexive saccades in visually healthy participants [24], [28]. Patients with amblyopia who have impaired retinal feedback mechanisms have deficits in the generation of secondary saccades [53], [54].

To our knowledge, this is the first study to investigate the role of retinal feedback in the generation of secondary saccades in an antisaccade task. To examine the relative contribution of extraretinal and retinal signals, we grouped secondary saccades according to their latency: short-latency (initiated <80 ms following the termination of the antisaccade, i.e., extraretinal; before retinal feedback can be used to program a saccade) and longer-latency (>80 ms) saccades, which can use the visual error signal derived from the position of the target image on the retina at the end of the primary saccade.

Our study unraveled that secondary saccades are the predominate strategy to improve accuracy in the antisaccade task, regardless of visual feedback. First, we found that the early-onset secondary saccades were generated independent of target feedback. In particular, these short latency secondary saccades were initiated with a six-fold higher frequency in the antisaccade task when compared to the reflexive saccade task. Second, secondary saccades in the no feedback condition were significantly more prevalent in the antisaccade task and their amplitude was associated with the extent of positional error at the end of the primary eye movement. Thus, we infer that extraretinal signals play a significant role in the generation of secondary eye movements in the antisaccade task.

Not surprisingly, target feedback enhanced the error correction process in the antisaccade task. Specifically, the amplitude and peak velocity of the late-onset secondary saccades depended on the target location, indicating that visual feedback improved the accuracy of the error correction. Our interpretation of these results is that retinal feedback is a significant factor in increasing the corrective role of secondary saccades. One potential caveat of our experimental paradigm is that the target was presented during the primary eye movement, therefore, secondary saccades in the feedback condition were most likely also influenced by the exogenous shift of attention towards the sudden onset of the target. Since our study did not include a condition that would allow us to study the generation of endogenously driven secondary saccades in the antisaccade task, our results cannot shed any light on the role of exogenous vs. endogenous factors in the generation of these saccades. As suggested by an anonymous reviewer, this could be accomplished in a future study by including a condition where static visual feedback was present at the antisaccade goal location, for example, by using a ‘placeholder’.

In conclusion, this study examined the contribution of online control during the primary saccade and offline control via secondary saccades in maintaining accuracy in the antisaccade task. We elicited large saccades and presented visual feedback at the spatial goal of the antisaccade to examine the role of visual feedback. Our data clearly show no effect of visual feedback on the primary eye movement online control. However, we report a distinct online control strategy in the antisaccade task which involves the extension of deceleration phase. Thus, once the antisaccade is initiated, the trajectory can be modulated via internal feedback loops. It remains to be determined whether the internal forward feedback model for antisaccades and reflexive saccade is mediated by the same neural substrate or whether different pathways are activated.
